# Supplementary material for: Sequencing of Argonaute-bound microRNA/mRNA hybrids reveals regulation of the unfolded protein response by microRNA-320a
Source: PLoS Genet. 2021 Dec 16;17(12):e1009934. doi: 10.1371/journal.pgen.1009934 (PMC8675727; doi:10.1371/journal.pgen.1009934)
Supplement: S1 Text — (PDF) [file pgen.1009934.s001.pdf]

SUPPLEMENTAL MATRIALS AND METHODS

Intermolecular ligation and library preparation for AGO-CLASH and qCLASH

Following RNase digestion, the beads were washed three times each with 1X PXL, 5X PXL (5X PBS, 0.1% SDS, 0.5% sodium deoxycholate, 0.5% NP-40), High-Stringency Buffer (15 mM Tris-HCL pH 7.5, 5 mM EDTA, 2.5 mM EGTA, 1% Triton X-100, 1% sodium deoxycholate, 0.1% SDS, 120 mM sodium chloride, 25 mM potassium chloride), High Salt Buffer (15 mM Tris-HCl pH 7.5, 5 mM EDTA, 2.5 mM EGTA, 1% Triton X-100, 1% sodium deoxycholate, 0.1% SDS, 1 M sodium chloride), and 1X PNK buffer (50 mM Tris-HCl pH 7.5, 10 mM magnesium chloride, 0.5% NP-40). Following washes, the samples were phosphorylated with 40U T4 PNK (NEB, M0201L) at 16°C for 40 minutes in 1X PNK Buffer, 80U of murine RNase Inhibitor, and 80 nmols of ATP in a total volume of 80 µL, washed three times with 1X PNK buffer, and intermolecular ligation performed with 500U T4 RNA Ligase I (NEB, M0437M) at 4°C overnight in 1X T4 RNA Ligase Buffer, 6% PEG8000, 10 mM KCl, 500U of murine RNase Inhibitor, and 500 nmols of ATP in a total volume of 500 µL. Beads were washed three times with 1X PNK buffer and dephosphorylated with 3U Calf Intestinal Alkaline Phosphatase (NEB, M0290) at 16°C for 40 minutes in 80U of murine RNase Inhibitor and 1X Cutsmart Buffer (NEB) in a total volume of 80 µL. Beads were washed twice with 1X PNK buffer-EGTA (50 mM Tris-HCl pH 7.5, 20 mM EGTA, 0.5% NP-40) and three times with 1X PNK buffer. For qCLASH, eighty pmols of RNA 3′ Adapter (RA3R) was ligated to the RNA with 80U of T4 RNA Ligase 2, truncated K227Q (NEB M0351L) in 1X T4 RNA Ligase Buffer, 10% PEG8000, and 80U of murine RNase Inhibitor (NEB, M0314L) in a total volume of 80 µL at 16°C overnight. CLASH samples were ligated with ^{32}P labeled RA3.

After 3′ adapter ligation, CLASH samples were washed three times with 1X PNK buffer and phosphorylated with 40U T4 PNK (NEB, M0201L) in 1X PNK Buffer, 80U of murine RNase Inhibitor, and 37.5 µCi ^{32}P-ATP at 37°C for 30 minutes with mixing. 80 nmoles of ATP was added and incubate for 15 minutes. The reaction was removed and washed with Hi-Stringency Buffer, Hi-Salt Buffer, 5X PXL Buffer, 1X PXL Buffer, and 1X PNK Buffer. Samples were resuspended in 1X NuPage Buffer, 100mM DTT diluted in PNK buffer and boiled at 75°C for 15 minutes, separated on a 4-12% NuPAGE gel, transferred to a nitrocellulose membrane, and then imaged on a Li-Cor Odyssey. Two regions corresponding to 100-125 and 125-150 kD were excised. qCLASH samples (to be processed without PAGE) were washed three times with 1X PNK buffer and protein/RNA complexes were eluted twice with 100 µL 100mM sodium bicarbonate and 1% sodium dodecyl sulfate at room temperature for 15 minutes. The two eluates are combined for RNA extraction.

Proteinase K was added to PK buffer (500 mM Tris-HCl pH 7.5, 250 mM sodium chloride, 50 mM EDTA) for a final concentration of 4 mg/mL. RNA was isolated from protein by adding 50 µL (qCLASH) or 160 µL (CLASH) of the diluted proteinase K mix to each sample at 37°C for 20 minutes. Phenol was

added to each reaction and incubated at room temperature for 8 minutes with 1400rpm of shaking. The samples were centrifuged at 18,000xg at 4°C for 10 minutes. The aqueous layer (~200 µL) was transferred to a new tube and 20 µL 3M NaOAc, 2 µL 15 mg/mL GlycoBlue (Invitrogen, AM9516), and 500 µL 1:1 mix of ethanol and isopropanol was added to each reaction and then incubated overnight at -20°C or -80°C for 1 hour. Following incubation, samples were centrifuged at 21,000xg at 4°C for 30 minutes and washed two times with 80% ethanol, centrifuging at 18,000xg at 4°C for 10 minutes. Samples were resuspended in nuclease-free water and phosphorylated with 10U of T4 PNK in reaction buffer (1X T4 PNK Ligase Buffer, 20U murine RNase Inhibitor, 15 nmols of ATP) in a volume of 15 µL at 16°C for 40 minutes. Reactions were purified twice by phenol:chloroform:isoamyl alcohol (Fisher, BP17521-400) (PCA), washed twice with chloroform and resuspended in 10 µL water. 100 pmols of RNA 5′ Adapter (RA5R for qCLASH and RA5 for CLASH) was ligated to the RNA with 10U T4 RNA Ligase in reaction buffer (1X T4 RNA Ligase Buffer, 2 µg of BSA, 20U murine RNase Inhibitor, 20 nmols of ATP) at 16°C overnight. The next day, samples were extracted with equal volume of PCA, vortexed at 1400 rpm for 8 minutes at room temperature, and centrifuged at 18,000xg for 10 minutes at room temperature. The aqueous layer (~200 µL) was transferred to a new tube, and 20 µL 3M NaOAc pH 5.2, 30 mg GlycoBlue (Invitrogen, AM9516), and 500 µL 1:1 mix of ethanol and isopropanol was added, and incubated at -80°C for 1 hour. Reactions were centrifuged at 21,000xg for 30 minutes, and the pellet was washed two times with 80% cold ethanol, and centrifuged at 18,000xg for 10 minutes after each wash. The pellet was allowed to dry and resuspended in RNase-free water.

To prepare cDNA libraries, RNA was incubated with 10 pmols each of RTP primer and dNTPs at 65°C for 5 minutes and reverse transcribed with 200U of SuperScript IV Reverse Transcriptase (Lifetech, 18090010) in 1X SuperScript RT Buffer, 100 nM DTT, and 40U murine RNase Inhibitor at 50°C in a reaction volume of 20 µL for 45 minutes, 55°C for 15 minutes, and 95°C for 5 minutes. To determine ideal PCR amplification for each library, 2% of the cDNA was PCR amplified for 10, 12, and 14 cycles with 0.08U Phusion High-Fidelity DNA Polymerase (NEB, M0530L), 1X Phusion HF buffer, 0.8 nmols dNTP mix, 2 pmols RP1, and 2 pmols RPI in a volume of 4 µL. Half the cDNA was used to generate the library using 3 cycles less than what was pre-determined with 1U Phusion High-Fidelity DNA Polymerase (NEB, M0530L), 1X Phusion HF buffer, 10 nmols dNTP mix, 25 pmols RP1, and 25 pmols RPI1 in a volume of 50 µL.
