# Supplementary figures and images for: Sequencing of Argonaute-bound microRNA/mRNA hybrids reveals regulation of the unfolded protein response by microRNA-320a
Source: PLoS Genet. 2021 Dec 16;17(12):e1009934. doi: 10.1371/journal.pgen.1009934 (PMC8675727; doi:10.1371/journal.pgen.1009934)

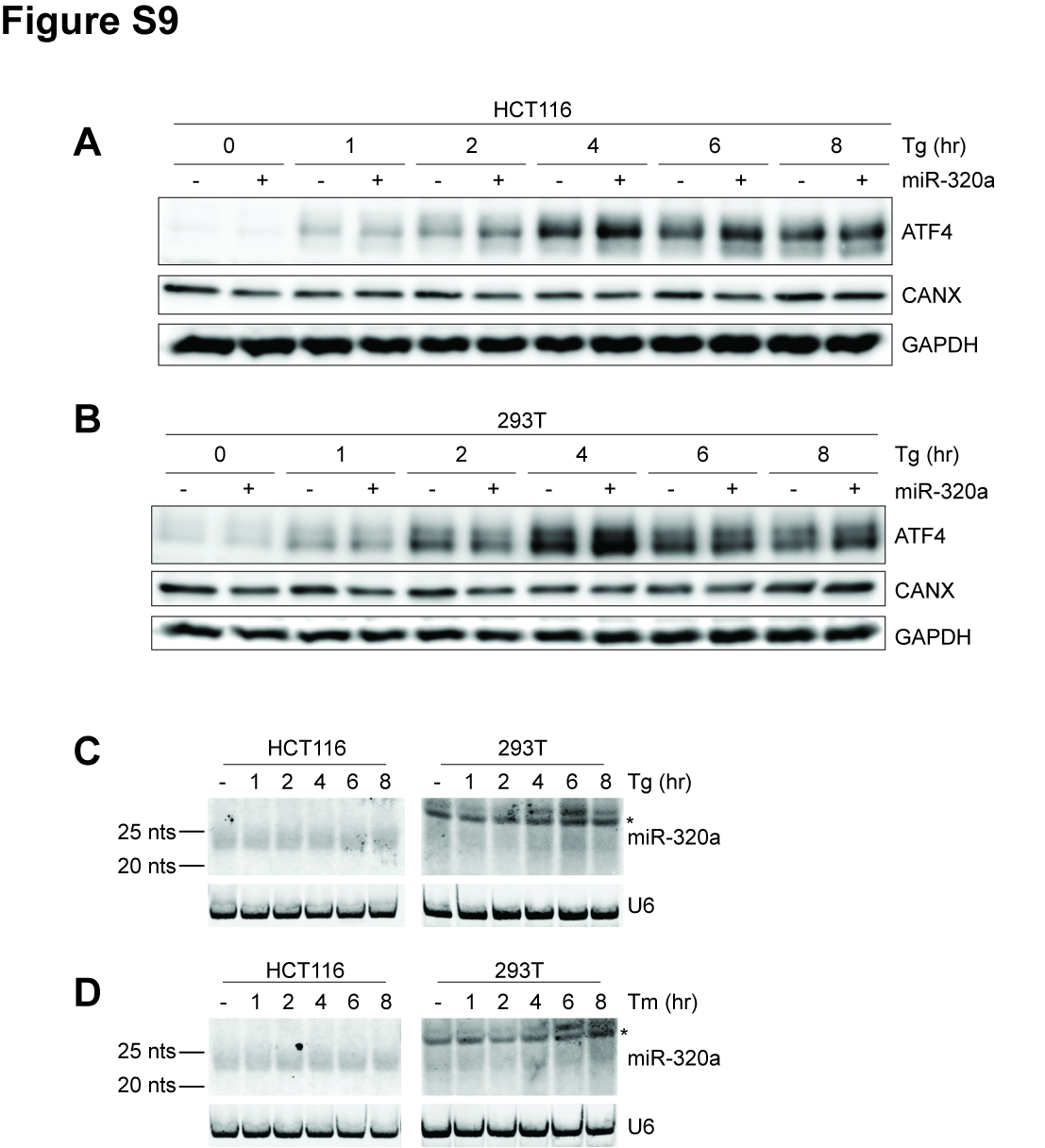

Supplement: S9 Fig — Western blot of ATF4, CANX and GAPDH in miR-320a mimic-transfected (A) HCT116 cells and (B) 293T cells treated with thapsigargin (Tg) for various times. irNorthern for miR-320a in HCT116 and 293T cells treated with (C) Tg and (D) tunicamycin (Tm). * marks a non-specific band detected by miR-320a probe in 293T cells. (TIF) [file pgen.1009934.s010.tif]
